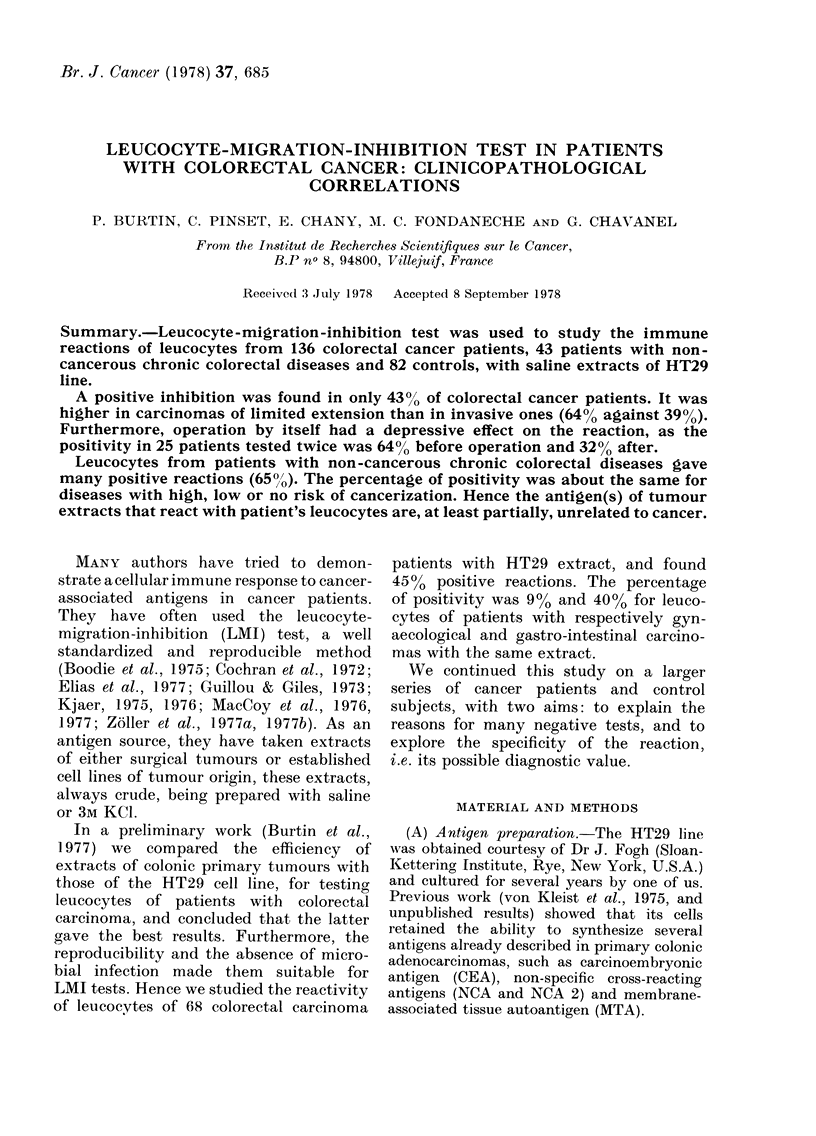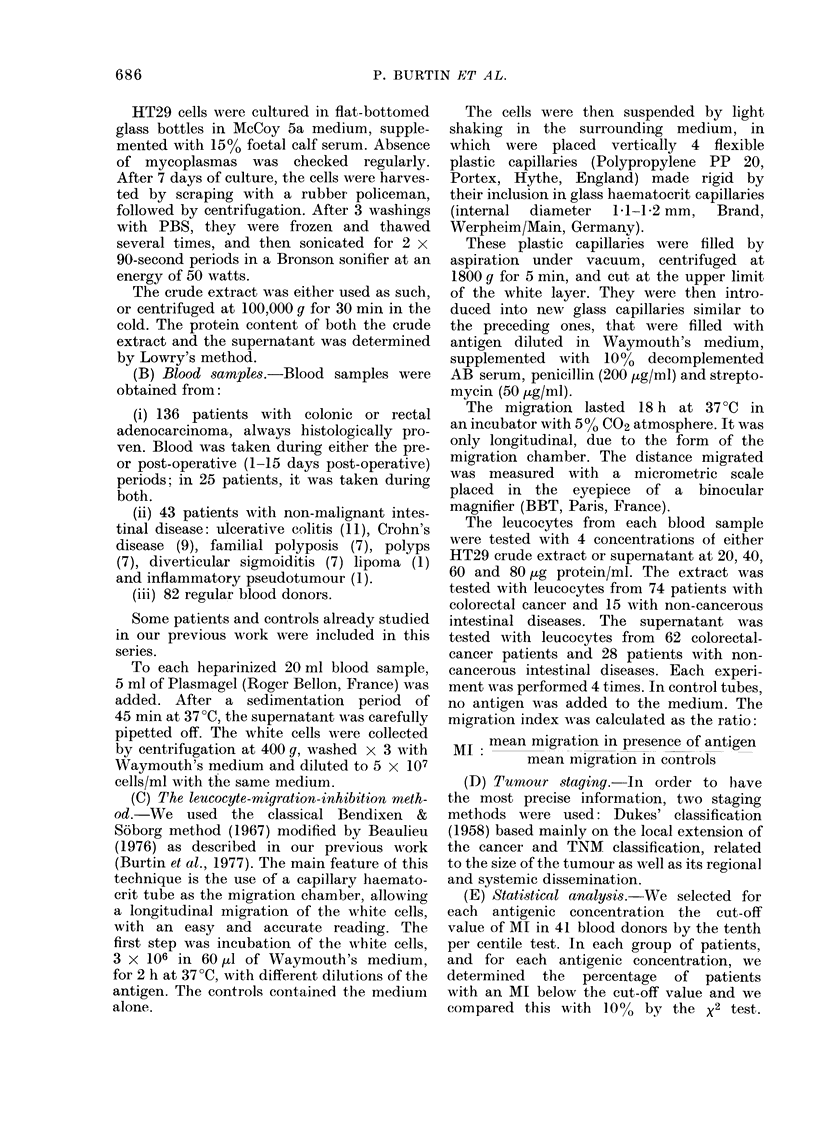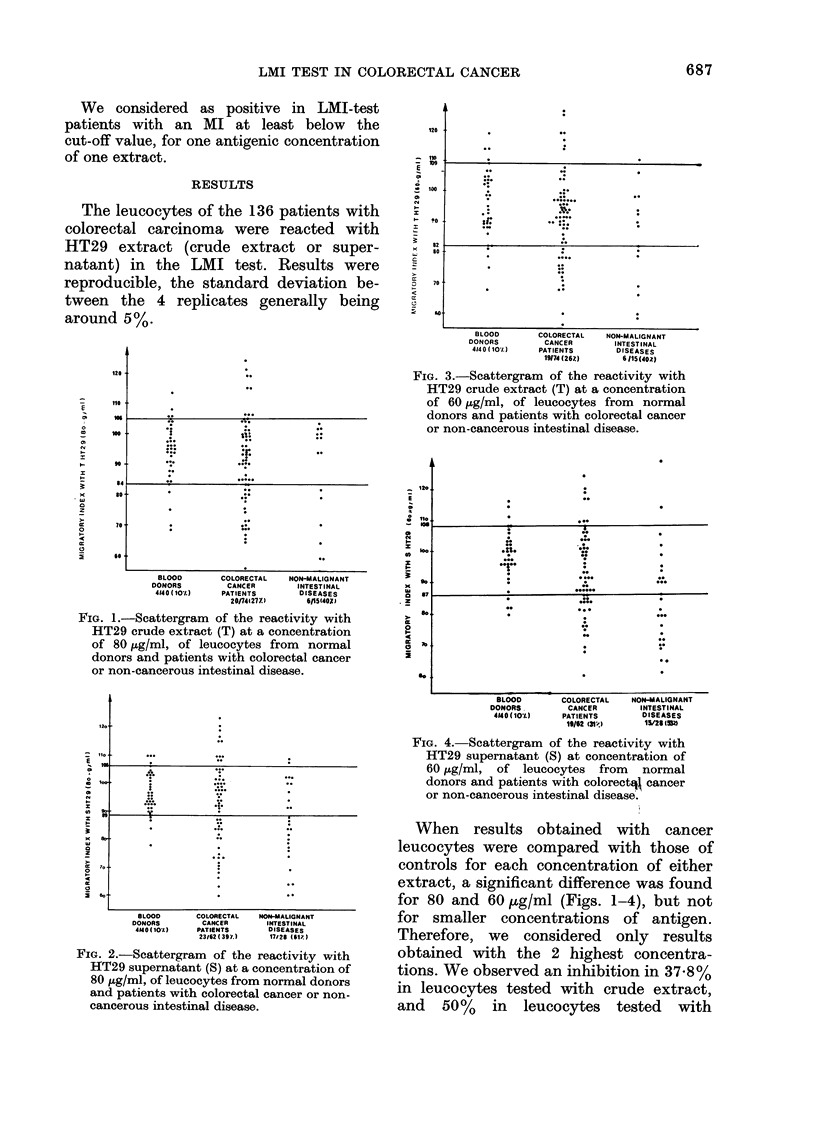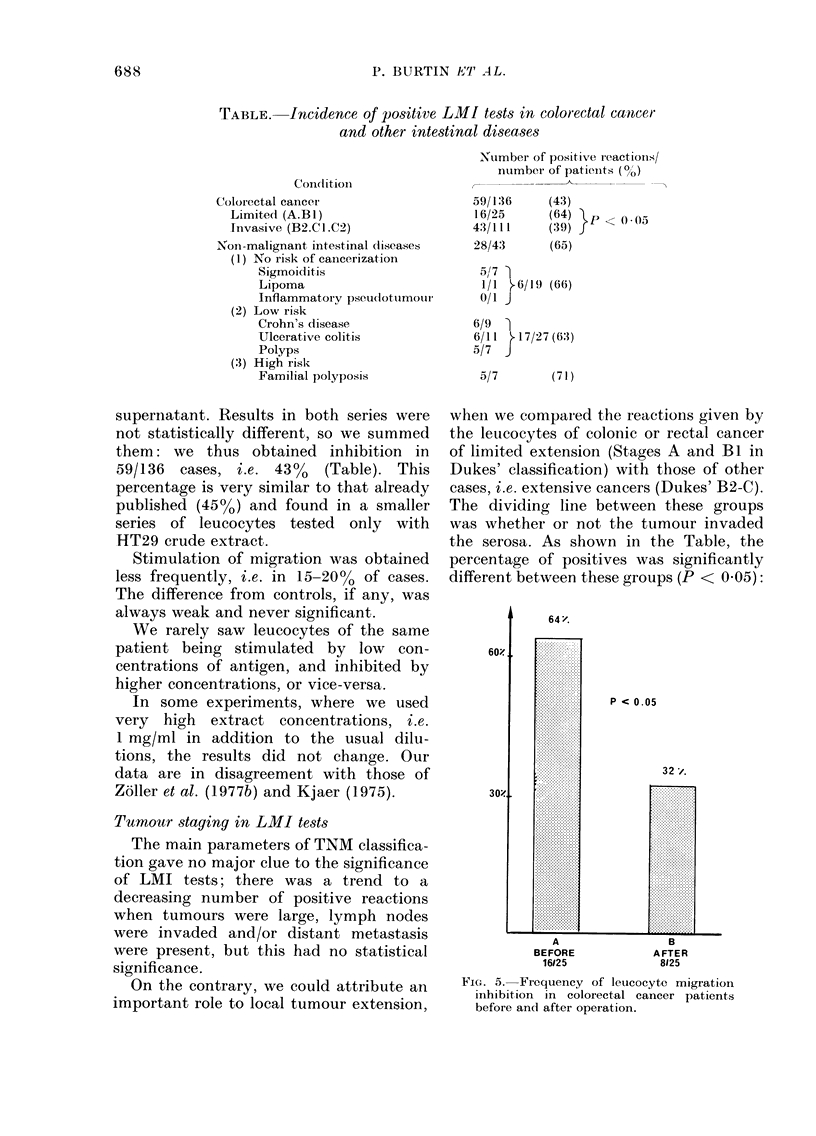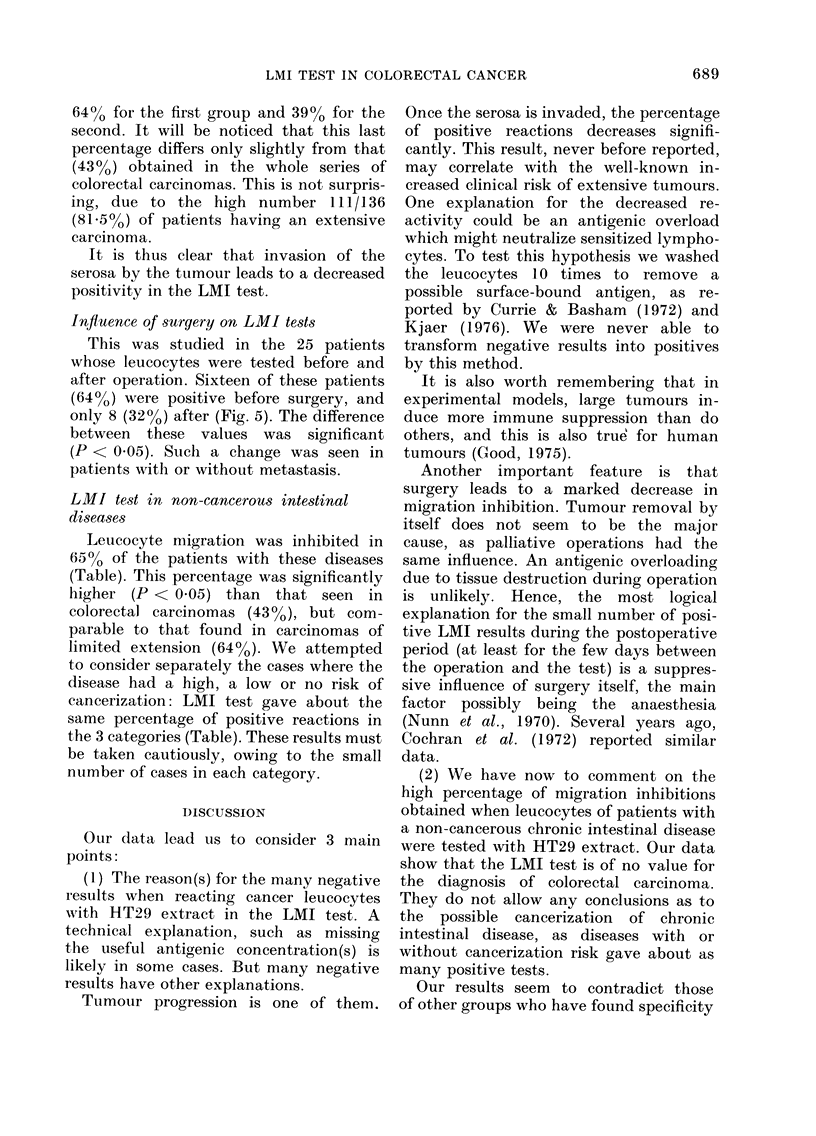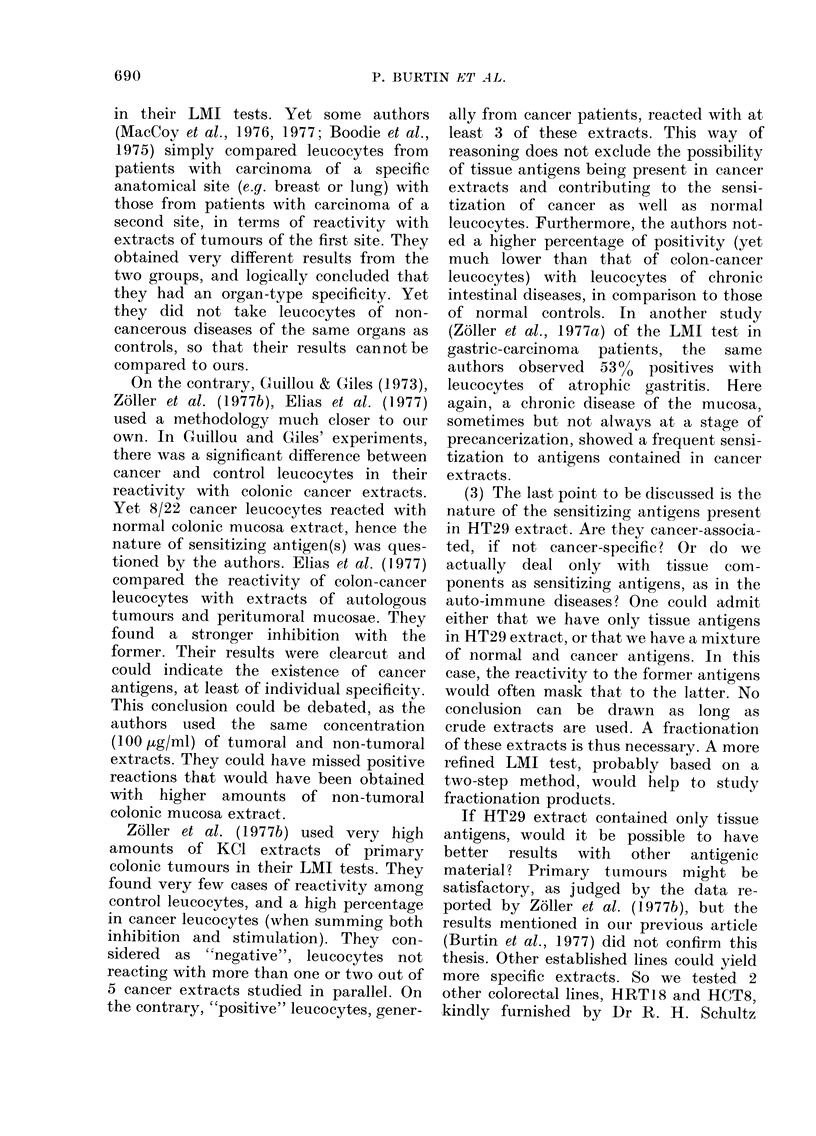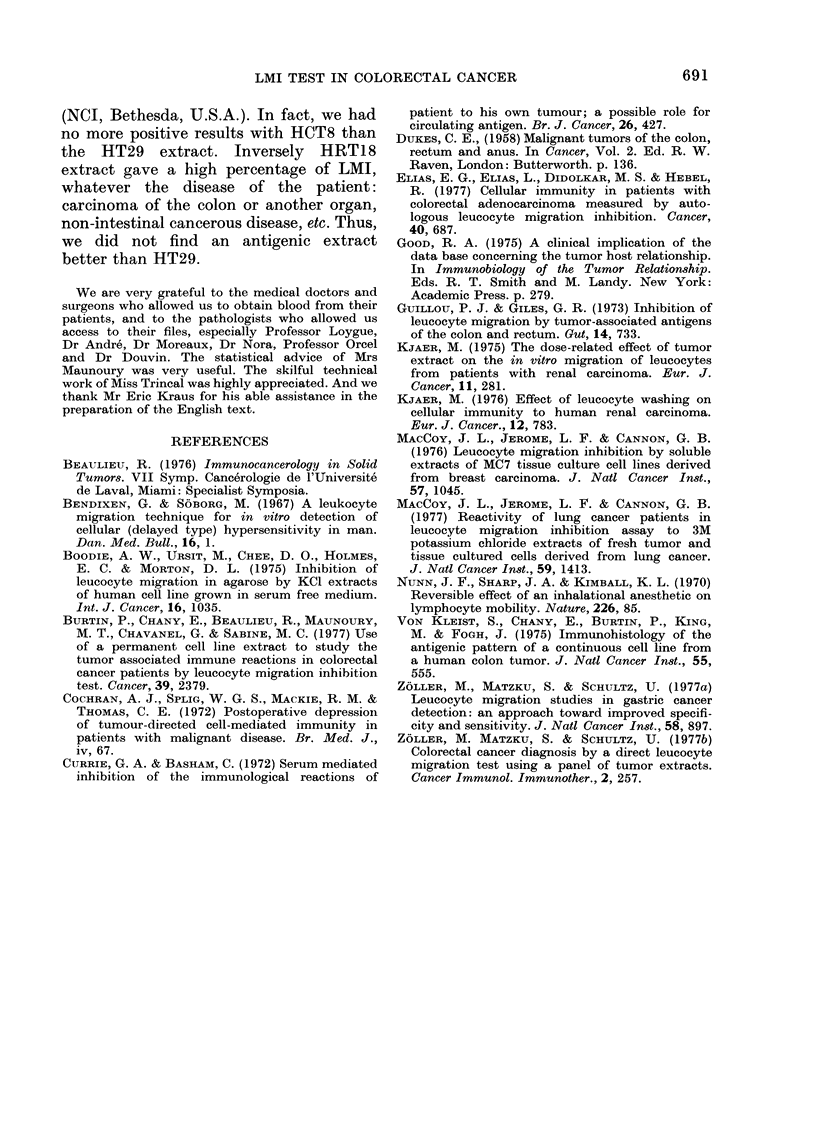# Leucocyte-migration-inhibition test in patients with colorectal cancer: clinicopathological correlations.

**DOI:** 10.1038/bjc.1978.273

**Published:** 1978-12

**Authors:** P. Burtin, C. Pinset, E. Chany, M. C. Fondaneche, G. Chavanel

## Abstract

Leucocyte-migration-inhibition test was used to study the immune reactions of leucocytes from 136 colorectal cancer patients, 43 patients with non-cancerous chronic colorectal diseases and 82 controls, with saline extracts of HT29 line. A positive inhibition was found in only 43% of colorectal cancer patients. It was higher in carcinomas of limited extension than in invasive ones (64% against 39%). Furthermore, operation by itself had a depressive effect on the reaction, as the positivity in 25 patients tested twice was 64% before operation and 32% after. Leucocytes from patients with non-cancerous chronic colorectal diseases gave many positive reactions (65%). The percentage of positivity was about the same for diseases with high, low or no risk of cancerization. Hence the antigen(s) of tumour extracts that react with patient's leucocytes are, at least partially, unrelated to cancer.


*Br. J. Cancer* (1978) **37**, 685

### LEUCOCYTE-MIGRATION-INHIBITION TEST IN PATIENTS WITH COLORECTAL CANCER: CLINICOPATHOLOGICAL CORRELATIONS

P. BURTIN, C. PINSET, E. CHANY, M. C. FONDANECHE AND G. CHAVANEL

*From the Institut de Recherches Scientifiques sur le Cancer, B.P n° 8, 94800, Villejuif, France*

Received 3 July 1978 Accepted 8 September 1978

**Summary.—Leucocyte-migration-inhibition test was used to study the immune reactions of leucocytes from 136 colorectal cancer patients, 43 patients with non-cancerous chronic colorectal diseases and 82 controls, with saline extracts of HT29 line.**

**A positive inhibition was found in only 43% of colorectal cancer patients. It was higher in carcinomas of limited extension than in invasive ones (64% against 39%). Furthermore, operation by itself had a depressive effect on the reaction, as the positivity in 25 patients tested twice was 64% before operation and 32% after.**

**Leucocytes from patients with non-cancerous chronic colorectal diseases gave many positive reactions (65%). The percentage of positivity was about the same for diseases with high, low or no risk of cancerization. Hence the antigen(s) of tumour extracts that react with patient's leucocytes are, at least partially, unrelated to cancer.**

MANY authors have tried to demonstrate a cellular immune response to cancer-associated antigens in cancer patients. They have often used the leucocyte-migration-inhibition (LMI) test, a well standardized and reproducible method (Boodie *et al.*, 1975; Cochran *et al.*, 1972; Elias *et al.*, 1977; Guillou & Giles, 1973; Kjaer, 1975, 1976; MacCoy *et al.*, 1976, 1977; Zöller *et al.*, 1977*a*, 1977*b*). As an antigen source, they have taken extracts of either surgical tumours or established cell lines of tumour origin, these extracts, always crude, being prepared with saline or 3M KCl.

In a preliminary work (Burtin *et al.*, 1977) we compared the efficiency of extracts of colonic primary tumours with those of the HT29 cell line, for testing leucocytes of patients with colorectal carcinoma, and concluded that the latter gave the best results. Furthermore, the reproducibility and the absence of microbial infection made them suitable for LMI tests. Hence we studied the reactivity of leucocytes of 68 colorectal carcinoma patients with HT29 extract, and found 45% positive reactions. The percentage of positivity was 9% and 40% for leucocytes of patients with respectively gynaecological and gastro-intestinal carcinomas with the same extract.

We continued this study on a larger series of cancer patients and control subjects, with two aims: to explain the reasons for many negative tests, and to explore the specificity of the reaction, *i.e.* its possible diagnostic value.

### MATERIAL AND METHODS

(A) *Antigen preparation.*—The HT29 line was obtained courtesy of Dr J. Fogh (Sloan-Kettering Institute, Rye, New York, U.S.A.) and cultured for several years by one of us. Previous work (von Kleist *et al.*, 1975, and unpublished results) showed that its cells retained the ability to synthesize several antigens already described in primary colonic adenocarcinomas, such as carcinoembryonic antigen (CEA), non-specific cross-reacting antigens (NCA and NCA 2) and membrane-associated tissue autoantigen (MTA).

HT29 cells were cultured in flat-bottomed glass bottles in McCoy 5a medium, supplemented with 15% foetal calf serum. Absence of mycoplasmas was checked regularly. After 7 days of culture, the cells were harvested by scraping with a rubber policeman, followed by centrifugation. After 3 washings with PBS, they were frozen and thawed several times, and then sonicated for 2 × 90-second periods in a Bronson sonifier at an energy of 50 watts.

The crude extract was either used as such, or centrifuged at 100,000 $g$ for 30 min in the cold. The protein content of both the crude extract and the supernatant was determined by Lowry's method.

(B) *Blood samples.*—Blood samples were obtained from:

(i) 136 patients with colonic or rectal adenocarcinoma, always histologically proven. Blood was taken during either the pre- or post-operative (1–15 days post-operative) periods; in 25 patients, it was taken during both.

(ii) 43 patients with non-malignant intestinal disease: ulcerative colitis (11), Crohn's disease (9), familial polyposis (7), polyps (7), diverticular sigmoiditis (7) lipoma (1) and inflammatory pseudotumour (1).

(iii) 82 regular blood donors.

Some patients and controls already studied in our previous work were included in this series.

To each heparinized 20 ml blood sample, 5 ml of Plasmagel (Roger Bellon, France) was added. After a sedimentation period of 45 min at 37 °C, the supernatant was carefully pipetted off. The white cells were collected by centrifugation at 400 $g$, washed × 3 with Waymouth's medium and diluted to $5 \times 10^7$ cells/ml with the same medium.

(C) *The leucocyte-migration-inhibition method.*—We used the classical Bendixen & Söborg method (1967) modified by Beaulieu (1976) as described in our previous work (Burtin *et al.*, 1977). The main feature of this technique is the use of a capillary haematocrit tube as the migration chamber, allowing a longitudinal migration of the white cells, with an easy and accurate reading. The first step was incubation of the white cells, $3 \times 10^6$ in 60 $\mu$l of Waymouth's medium, for 2 h at 37 °C, with different dilutions of the antigen. The controls contained the medium alone.

The cells were then suspended by light shaking in the surrounding medium, in which were placed vertically 4 flexible plastic capillaries (Polypropylene PP 20, Portex, Hythe, England) made rigid by their inclusion in glass haematocrit capillaries (internal diameter 1·1–1·2 mm, Brand, Werpheim/Main, Germany).

These plastic capillaries were filled by aspiration under vacuum, centrifuged at 1800 $g$ for 5 min, and cut at the upper limit of the white layer. They were then introduced into new glass capillaries similar to the preceding ones, that were filled with antigen diluted in Waymouth's medium, supplemented with 10% decomplemented AB serum, penicillin (200 $\mu$g/ml) and streptomycin (50 $\mu$g/ml).

The migration lasted 18 h at 37 °C in an incubator with 5% $CO_2$ atmosphere. It was only longitudinal, due to the form of the migration chamber. The distance migrated was measured with a micrometric scale placed in the eyepiece of a binocular magnifier (BBT, Paris, France).

The leucocytes from each blood sample were tested with 4 concentrations of either HT29 crude extract or supernatant at 20, 40, 60 and 80 $\mu$g protein/ml. The extract was tested with leucocytes from 74 patients with colorectal cancer and 15 with non-cancerous intestinal diseases. The supernatant was tested with leucocytes from 62 colorectal-cancer patients and 28 patients with non-cancerous intestinal diseases. Each experiment was performed 4 times. In control tubes, no antigen was added to the medium. The migration index was calculated as the ratio:

$$\text{MI} : \frac{\text{mean migration in presence of antigen}}{\text{mean migration in controls}}$$

(D) *Tumour staging.*—In order to have the most precise information, two staging methods were used: Dukes' classification (1958) based mainly on the local extension of the cancer and TNM classification, related to the size of the tumour as well as its regional and systemic dissemination.

(E) *Statistical analysis.*—We selected for each antigenic concentration the cut-off value of MI in 41 blood donors by the tenth per centile test. In each group of patients, and for each antigenic concentration, we determined the percentage of patients with an MI below the cut-off value and we compared this with 10% by the $\chi^2$ test.

We considered as positive in LMI-test patients with an MI at least below the cut-off value, for one antigenic concentration of one extract.

### RESULTS

The leucocytes of the 136 patients with colorectal carcinoma were reacted with HT29 extract (crude extract or supernatant) in the LMI test. Results were reproducible, the standard deviation between the 4 replicates generally being around 5%.

FIG. 1.—Scattergram of the reactivity with HT29 crude extract (T) at a concentration of 80 $\mu$g/ml, of leucocytes from normal donors and patients with colorectal cancer or non-cancerous intestinal disease.

FIG. 2.—Scattergram of the reactivity with HT29 supernatant (S) at a concentration of 80 $\mu$g/ml, of leucocytes from normal donors and patients with colorectal cancer or non-cancerous intestinal disease.

FIG. 3.—Scattergram of the reactivity with HT29 crude extract (T) at a concentration of 60 $\mu$g/ml, of leucocytes from normal donors and patients with colorectal cancer or non-cancerous intestinal disease.

FIG. 4.—Scattergram of the reactivity with HT29 supernatant (S) at concentration of 60 $\mu$g/ml, of leucocytes from normal donors and patients with colorectal cancer or non-cancerous intestinal disease.

When results obtained with cancer leucocytes were compared with those of controls for each concentration of either extract, a significant difference was found for 80 and 60 $\mu$g/ml (Figs. 1–4), but not for smaller concentrations of antigen. Therefore, we considered only results obtained with the 2 highest concentrations. We observed an inhibition in 37·8% in leucocytes tested with crude extract, and 50% in leucocytes tested with

TABLE.—*Incidence of positive LMI tests in colorectal cancer and other intestinal diseases*

| Condition | Number of positive reactions/ number of patients (%) | | |
|---|---|---|---|
| Colorectal cancer | 59/136 | (43) | |
| Limited (A.B1) | 16/25 | (64) | $P < 0{\cdot}05$ |
| Invasive (B2.C1.C2) | 43/111 | (39) | |
| Non-malignant intestinal diseases | 28/43 | (65) | |
| (1) No risk of cancerization | | | |
| Sigmoiditis | 5/7 | 6/19 (66) | |
| Lipoma | 1/1 | | |
| Inflammatory pseudotumour | 0/1 | | |
| (2) Low risk | | | |
| Crohn's disease | 6/9 | 17/27 (63) | |
| Ulcerative colitis | 6/11 | | |
| Polyps | 5/7 | | |
| (3) High risk | | | |
| Familial polyposis | 5/7 | (71) | |

supernatant. Results in both series were not statistically different, so we summed them: we thus obtained inhibition in 59/136 cases, *i.e.* 43% (Table). This percentage is very similar to that already published (45%) and found in a smaller series of leucocytes tested only with HT29 crude extract.

Stimulation of migration was obtained less frequently, *i.e.* in 15–20% of cases. The difference from controls, if any, was always weak and never significant.

We rarely saw leucocytes of the same patient being stimulated by low concentrations of antigen, and inhibited by higher concentrations, or vice-versa.

In some experiments, where we used very high extract concentrations, *i.e.* 1 mg/ml in addition to the usual dilutions, the results did not change. Our data are in disagreement with those of Zöller *et al.* (1977*b*) and Kjaer (1975).

*Tumour staging in LMI tests*

The main parameters of TNM classification gave no major clue to the significance of LMI tests; there was a trend to a decreasing number of positive reactions when tumours were large, lymph nodes were invaded and/or distant metastasis were present, but this had no statistical significance.

On the contrary, we could attribute an important role to local tumour extension, when we compared the reactions given by the leucocytes of colonic or rectal cancer of limited extension (Stages A and B1 in Dukes' classification) with those of other cases, *i.e.* extensive cancers (Dukes' B2-C). The dividing line between these groups was whether or not the tumour invaded the serosa. As shown in the Table, the percentage of positives was significantly different between these groups ($P < 0{\cdot}05$):

FIG. 5.—Frequency of leucocyte migration inhibition in colorectal cancer patients before and after operation.

64% for the first group and 39% for the second. It will be noticed that this last percentage differs only slightly from that (43%) obtained in the whole series of colorectal carcinomas. This is not surprising, due to the high number 111/136 (81·5%) of patients having an extensive carcinoma.

It is thus clear that invasion of the serosa by the tumour leads to a decreased positivity in the LMI test.

*Influence of surgery on LMI tests*

This was studied in the 25 patients whose leucocytes were tested before and after operation. Sixteen of these patients (64%) were positive before surgery, and only 8 (32%) after (Fig. 5). The difference between these values was significant ($P < 0{\cdot}05$). Such a change was seen in patients with or without metastasis.

*LMI test in non-cancerous intestinal diseases*

Leucocyte migration was inhibited in 65% of the patients with these diseases (Table). This percentage was significantly higher ($P < 0{\cdot}05$) than that seen in colorectal carcinomas (43%), but comparable to that found in carcinomas of limited extension (64%). We attempted to consider separately the cases where the disease had a high, a low or no risk of cancerization: LMI test gave about the same percentage of positive reactions in the 3 categories (Table). These results must be taken cautiously, owing to the small number of cases in each category.

### DISCUSSION

Our data lead us to consider 3 main points:

(1) The reason(s) for the many negative results when reacting cancer leucocytes with HT29 extract in the LMI test. A technical explanation, such as missing the useful antigenic concentration(s) is likely in some cases. But many negative results have other explanations.

Tumour progression is one of them. Once the serosa is invaded, the percentage of positive reactions decreases significantly. This result, never before reported, may correlate with the well-known increased clinical risk of extensive tumours. One explanation for the decreased reactivity could be an antigenic overload which might neutralize sensitized lymphocytes. To test this hypothesis we washed the leucocytes 10 times to remove a possible surface-bound antigen, as reported by Currie & Basham (1972) and Kjaer (1976). We were never able to transform negative results into positives by this method.

It is also worth remembering that in experimental models, large tumours induce more immune suppression than do others, and this is also true for human tumours (Good, 1975).

Another important feature is that surgery leads to a marked decrease in migration inhibition. Tumour removal by itself does not seem to be the major cause, as palliative operations had the same influence. An antigenic overloading due to tissue destruction during operation is unlikely. Hence, the most logical explanation for the small number of positive LMI results during the postoperative period (at least for the few days between the operation and the test) is a suppressive influence of surgery itself, the main factor possibly being the anaesthesia (Nunn *et al.*, 1970). Several years ago, Cochran *et al.* (1972) reported similar data.

(2) We have now to comment on the high percentage of migration inhibitions obtained when leucocytes of patients with a non-cancerous chronic intestinal disease were tested with HT29 extract. Our data show that the LMI test is of no value for the diagnosis of colorectal carcinoma. They do not allow any conclusions as to the possible cancerization of chronic intestinal disease, as diseases with or without cancerization risk gave about as many positive tests.

Our results seem to contradict those of other groups who have found specificity in their LMI tests. Yet some authors (MacCoy *et al.*, 1976, 1977; Boodie *et al.*, 1975) simply compared leucocytes from patients with carcinoma of a specific anatomical site (*e.g.* breast or lung) with those from patients with carcinoma of a second site, in terms of reactivity with extracts of tumours of the first site. They obtained very different results from the two groups, and logically concluded that they had an organ-type specificity. Yet they did not take leucocytes of non-cancerous diseases of the same organs as controls, so that their results cannot be compared to ours.

On the contrary, Guillou & Giles (1973), Zöller *et al.* (1977*b*), Elias *et al.* (1977) used a methodology much closer to our own. In Guillou and Giles' experiments, there was a significant difference between cancer and control leucocytes in their reactivity with colonic cancer extracts. Yet 8/22 cancer leucocytes reacted with normal colonic mucosa extract, hence the nature of sensitizing antigen(s) was questioned by the authors. Elias *et al.* (1977) compared the reactivity of colon-cancer leucocytes with extracts of autologous tumours and peritumoral mucosae. They found a stronger inhibition with the former. Their results were clearcut and could indicate the existence of cancer antigens, at least of individual specificity. This conclusion could be debated, as the authors used the same concentration (100 $\mu$g/ml) of tumoral and non-tumoral extracts. They could have missed positive reactions that would have been obtained with higher amounts of non-tumoral colonic mucosa extract.

Zöller *et al.* (1977*b*) used very high amounts of KCl extracts of primary colonic tumours in their LMI tests. They found very few cases of reactivity among control leucocytes, and a high percentage in cancer leucocytes (when summing both inhibition and stimulation). They considered as "negative", leucocytes not reacting with more than one or two out of 5 cancer extracts studied in parallel. On the contrary, "positive" leucocytes, generally from cancer patients, reacted with at least 3 of these extracts. This way of reasoning does not exclude the possibility of tissue antigens being present in cancer extracts and contributing to the sensitization of cancer as well as normal leucocytes. Furthermore, the authors noted a higher percentage of positivity (yet much lower than that of colon-cancer leucocytes) with leucocytes of chronic intestinal diseases, in comparison to those of normal controls. In another study (Zöller *et al.*, 1977*a*) of the LMI test in gastric-carcinoma patients, the same authors observed 53% positives with leucocytes of atrophic gastritis. Here again, a chronic disease of the mucosa, sometimes but not always at a stage of precancerization, showed a frequent sensitization to antigens contained in cancer extracts.

(3) The last point to be discussed is the nature of the sensitizing antigens present in HT29 extract. Are they cancer-associated, if not cancer-specific? Or do we actually deal only with tissue components as sensitizing antigens, as in the auto-immune diseases? One could admit either that we have only tissue antigens in HT29 extract, or that we have a mixture of normal and cancer antigens. In this case, the reactivity to the former antigens would often mask that to the latter. No conclusion can be drawn as long as crude extracts are used. A fractionation of these extracts is thus necessary. A more refined LMI test, probably based on a two-step method, would help to study fractionation products.

If HT29 extract contained only tissue antigens, would it be possible to have better results with other antigenic material? Primary tumours might be satisfactory, as judged by the data reported by Zöller *et al.* (1977*b*), but the results mentioned in our previous article (Burtin *et al.*, 1977) did not confirm this thesis. Other established lines could yield more specific extracts. So we tested 2 other colorectal lines, HRT18 and HCT8, kindly furnished by Dr R. H. Schultz

(NCI, Bethesda, U.S.A.). In fact, we had no more positive results with HCT8 than the HT29 extract. Inversely HRT18 extract gave a high percentage of LMI, whatever the disease of the patient: carcinoma of the colon or another organ, non-intestinal cancerous disease, *etc*. Thus, we did not find an antigenic extract better than HT29.

We are very grateful to the medical doctors and surgeons who allowed us to obtain blood from their patients, and to the pathologists who allowed us access to their files, especially Professor Loygue, Dr André, Dr Moreaux, Dr Nora, Professor Orcel and Dr Douvin. The statistical advice of Mrs Maunoury was very useful. The skilful technical work of Miss Trincal was highly appreciated. And we thank Mr Eric Kraus for his able assistance in the preparation of the English text.

### REFERENCES

BEAULIEU, R. (1976) *Immunocancerology in Solid Tumors*. VII Symp. Cancérologie de l'Université de Laval, Miami: Specialist Symposia.

BENDIXEN, G. & SÖBORG, M. (1967) A leukocyte migration technique for *in vitro* detection of cellular (delayed type) hypersensitivity in man. *Dan. Med. Bull.*, **16**, 1.

BOODIE, A. W., URSIT, M., CHEE, D. O., HOLMES, E. C. & MORTON, D. L. (1975) Inhibition of leucocyte migration in agarose by KCl extracts of human cell line grown in serum free medium. *Int. J. Cancer*, **16**, 1035.

BURTIN, P., CHANY, E., BEAULIEU, R., MAUNOURY, M. T., CHAVANEL, G. & SABINE, M. C. (1977) Use of a permanent cell line extract to study the tumor associated immune reactions in colorectal cancer patients by leucocyte migration inhibition test. *Cancer*, **39**, 2379.

COCHRAN, A. J., SPLIG, W. G. S., MACKIE, R. M. & THOMAS, C. E. (1972) Postoperative depression of tumour-directed cell-mediated immunity in patients with malignant disease. *Br. Med. J.*, iv, 67.

CURRIE, G. A. & BASHAM, C. (1972) Serum mediated inhibition of the immunological reactions of patient to his own tumour; a possible role for circulating antigen. *Br. J. Cancer*, **26**, 427.

DUKES, C. E., (1958) Malignant tumors of the colon, rectum and anus. In *Cancer*, Vol. 2. Ed. R. W. Raven, London: Butterworth. p. 136.

ELIAS, E. G., ELIAS, L., DIDOLKAR, M. S. & HEBEL, R. (1977) Cellular immunity in patients with colorectal adenocarcinoma measured by autologous leucocyte migration inhibition. *Cancer*, **40**, 687.

GOOD, R. A. (1975) A clinical implication of the data base concerning the tumor host relationship. In *Immunobiology of the Tumor Relationship.* Eds. R. T. Smith and M. Landy. New York: Academic Press. p. 279.

GUILLOU, P. J. & GILES, G. R. (1973) Inhibition of leucocyte migration by tumor-associated antigens of the colon and rectum. *Gut*, **14**, 733.

KJAER, M. (1975) The dose-related effect of tumor extract on the *in vitro* migration of leucocytes from patients with renal carcinoma. *Eur. J. Cancer*, **11**, 281.

KJAER, M. (1976) Effect of leucocyte washing on cellular immunity to human renal carcinoma. *Eur. J. Cancer.*, **12**, 783.

MACCOY, J. L., JEROME, L. F. & CANNON, G. B. (1976) Leucocyte migration inhibition by soluble extracts of MC7 tissue culture cell lines derived from breast carcinoma. *J. Natl Cancer Inst.*, **57**, 1045.

MACCOY, J. L., JEROME, L. F. & CANNON, G. B. (1977) Reactivity of lung cancer patients in leucocyte migration inhibition assay to 3M potassium chloride extracts of fresh tumor and tissue cultured cells derived from lung cancer. *J. Natl Cancer Inst.*, **59**, 1413.

NUNN, J. F., SHARP, J. A. & KIMBALL, K. L. (1970) Reversible effect of an inhalational anesthetic on lymphocyte mobility. *Nature*, **226**, 85.

VON KLEIST, S., CHANY, E., BURTIN, P., KING, M. & FOGH, J. (1975) Immunohistology of the antigenic pattern of a continuous cell line from a human colon tumor. *J. Natl Cancer Inst.*, **55**, 555.

ZÖLLER, M., MATZKU, S. & SCHULTZ, U. (1977*a*) Leucocyte migration studies in gastric cancer detection: an approach toward improved specificity and sensitivity. *J. Natl Cancer Inst.*, **58**, 897.

ZÖLLER, M. MATZKU, S. & SCHULTZ, U. (1977*b*) Colorectal cancer diagnosis by a direct leucocyte migration test using a panel of tumor extracts. *Cancer Immunol. Immunother.*, **2**, 257.